# Simplifying the Animal Welfare Assessment Grid for enhanced accessibility

**DOI:** 10.3389/fvets.2024.1459560

**Published:** 2024-11-19

**Authors:** Ji-yoon Kim, Jae-Hyeon Choi, HyunYoung Ryu, Hye-Jin Kang

**Affiliations:** Department of Horse/Companion and Wild Animals, College of Ecology and Environmental Science, Kyungpook National University, Sangju, Republic of Korea

**Keywords:** animal welfare, animal welfare assessment, Animal Welfare Assessment Grid, Five Domains Model, zoo animal welfare, observation-based welfare assessment, simplified welfare assessment, non-expert welfare evaluation

## Abstract

Ensuring animal welfare is essential for both the well-being of zoo animals and the effective management of zoological facilities. This study introduces the Simplified Animal Welfare Assessment Grid (S-AWAG), a streamlined adaptation of the original AWAG framework that integrates the Five Domains Model with an observation-based approach. Designed for non-expert users, S-AWAG focuses on easily observable welfare indicators—such as health and environmental conditions—making it particularly suitable for small, private zoos, including petting zoos, roadside zoos, indoor zoos, and animal cafés. We conducted welfare assessments on 304 animals from 11 species across nine zoos in South Korea. The results revealed significant differences in welfare standards between accredited and non-accredited zoos, with accredited facilities consistently demonstrating better welfare conditions (*p* < 0.05). The tool exhibited high inter-rater reliability (IRR = 0.839), confirming its consistency across assessors with varying levels of expertise and ensuring reliable and accurate results. Pearson correlation analysis identified strong positive associations between health and environmental factors, reinforcing the comprehensive nature of the tool’s evaluation approach. With its user-friendly, efficient, and adaptable design, S-AWAG has the potential to improve animal welfare standards not only in South Korea but also globally, particularly in smaller, resource-constrained facilities.

## 1 Introduction

In modern society, zoos serve four primary roles: conservation, research, recreation, and education (1–3). The integration of animal ecology and behavioral research into conservation efforts has elevated scientific practices aimed at enhancing animal welfare (1–4). These activities provide the public with opportunities to learn about wildlife, thereby increasing awareness of the importance of nature conservation (1, 2, 4). However, the role of zoos has evolved significantly over time. In 19th-century Europe, zoos were symbols of political power and wealth (2–4). During this period, limited understanding of animals’ cognitive abilities and emotions led to their confinement in unnatural, restricted environments solely for public entertainment (3–6). Advances in research on animal cognition, sensory experiences, and sentience—the capacity to experience pleasure and pain—significantly raised awareness of animal welfare (5–7). This shift profoundly impacted animal welfare policies and zoo management practices, leading to the enactment of laws such as the Animal Welfare Act,^1^ which facilitated global changes aimed at legally protecting animal welfare (4, 5, 7).^2^^,^^3^^,^^4^^,^^5^ International non-profit organizations like the Association of Zoos and Aquariums (AZA)^6^ and the European Association of Zoos and Aquaria (EAZA)^7^ have also played crucial roles in establishing global animal welfare standards (6–8). These organizations promote improved animal care and encourage zoos worldwide to adhere to these standards (4, 6, 8). Consequently, modern zoos have shifted from merely ensuring the physical safety of animals to providing environments that allow animals to exhibit their natural behaviors (1, 2, 4).

South Korea has similarly benchmarked these international standards to enhance both animal welfare assessments and overall zoo management (9–11). The enactment of the 2016 Act on the Management of Zoos and Aquariums^8^ marked a significant advancement in the legal and institutional frameworks related to animal welfare (12, 13). This law introduced a registration system to ensure that all animal exhibition facilities met specific criteria. However, the initial version of the law had limitations, particularly its lack of clear criteria for managing the welfare of small-scale, non-accredited zoos—including petting zoos, indoor zoos, roadside zoos, and animal cafés (9, 12, 13). To address these issues, the 2022 amendment^9^ strengthened the licensing system, mandated regular inspections and welfare assessments, and enforced stricter penalties, including the closure of facilities that failed to meet welfare standards.^10^^,^^11^

Despite these legal and institutional reforms, more than 80% of small private zoos in South Korea^12^ continue to exhibit inadequate welfare standards (11, 13). This is largely due to insufficient financial resources, a lack of professional staff, and weak legal regulations and oversight (14–16). Unlike large public zoos, small private zoos—especially those focused on entertainment and commercial purposes—tend to prioritize profit over animal welfare (5, 17, 18). This operational approach often results in minimal staff training, leaving personnel unprepared to fully comprehend or implement complex welfare assessment standards, which leads to issues during keeper-based assessments (15, 16, 19). Consequently, inadequate environments frequently result in animal deaths, widely reported in the media and exacerbating negative public perceptions^13^^,^^14^. In such a climate, some private zoos tend to avoid external welfare assessments^15^^,^^16^, delaying accurate evaluation and necessary improvements to animal welfare (8, 15, 20). Furthermore, while large zoos have the resources to implement internal monitoring systems and welfare improvement programs, smaller zoos often lack the equipment and data analysis capabilities needed for regular and systematic monitoring (16, 19, 21). This lack of resources leads small-scale zoos to rely passively on legal regulations or external assessments rather than voluntarily making efforts to improve welfare (6, 8, 20). Volunteers and NGOs concerned with animal welfare often actively engage in improving conditions in small zoos, but they too frequently lack systematic training and field experience, highlighting practical limitations in welfare management (20, 22, 23).

To address these challenges a simplified animal welfare assessment tool is urgently needed—one that can be reliably applied across different types and sizes of zoos, even by individuals from diverse backgrounds. Particularly in resource-constrained small zoos, a standardized tool that can be utilized without expert supervision is essential (14–16). In response to this need, we developed the Simplified Animal Welfare Assessment Grid (S-AWAG), designed to improve the efficiency and accessibility of welfare assessments and facilitate continuous welfare improvements. S-AWAG allows non-experts to participate in assessments, incorporating diverse perspectives and enhancing objectivity (23–25). While experts may possess a deep understanding of specific species or environments, their evaluations can sometimes introduce bias (25–27). Non-experts, on the other hand, provide fresh perspectives from diverse backgrounds, contributing to a more comprehensive and balanced analysis (19, 22, 28). Furthermore, public involvement in the evaluation process enhances transparency and promotes regular assessments and external oversight (25, 27, 29). This transparency helps maintain zoo welfare standards in the public eye, motivating operators to maintain or improve conditions and reducing the likelihood of avoiding external evaluations (14, 20, 24). Broader participation also facilitates large-scale monitoring, enabling quicker assessments and more rapid welfare improvements (6, 25, 29). Additionally, the involvement of non-experts could help reduce costs by minimizing the need for highly paid experts, streamlining evaluation procedures, and lowering equipment and resource requirements through tools like S-AWAG (21, 26, 30). As a result, zoo operators can engage more actively in welfare improvements (8, 20, 25).

Based on these considerations, we hypothesize that S-AWAG will prove more effective and efficient than existing tools in enabling non-experts to assess animal welfare. To validate this, we applied S-AWAG in various zoo environments to evaluate its reliability and validity, with a particular focus on its effectiveness in improving welfare standards in small, non-accredited zoos where non-experts play a major role.

## 2 Materials and methods

### 2.1 Researchers

This study was conducted by three students from the Department of Horse, Companion, and Wildlife Sciences at Kyungpook National University. During the second semester of 2023, they completed a course titled Animal Welfare Studies, where they gained theoretical knowledge of various welfare assessment methods and conducted in-depth studies on domestic and international laws and policies related to animal welfare. Utilizing resources from World Animal Protection^17^, they explored animal rights, ethics, and the Five Freedoms.^18^ Based on this knowledge, the students engaged in case studies and discussions about various animal welfare issues, including zoo animals, animal testing, farm animals, and animal shows.

From November 2022 to January 2023, the research team visited nine zoos across South Korea to collect data. To ensure objectivity and consistency, they received pre-training from an expert on the use of the Animal Welfare Assessment Grid (AWAG) (17–19). This training included practical exercises and case studies to familiarize the team with the assessment procedures. Through this training, the researchers learned to assess animals’ physical and psychological conditions, evaluating whether the environments allowed for the expression of instinctive behaviors and whether the animals were experiencing distress in inappropriate conditions.

As part of their preparation, the team conducted preliminary welfare assessments at one Group A zoo and a poorly managed public zoo (not included in the study). During these visits, they assessed animals such as Eurasian eagle-owls (*Bubo bubo*), eagles (*Aquila* spp.), blue-and-yellow macaws (*Ara ararauna*), red-and-green macaws (*Ara chloropterus*), and rabbits (*Oryctolagus cuniculus*). These preliminary assessments allowed the team to test their tools and procedures in real-world scenarios and verify the appropriateness of their evaluation methods (26, 31, 32). Observing behavioral indicators like stereotypic behaviors helped them understand the impact of adverse environments on animals, thereby enhancing their practical skills in assessing animal welfare levels.

Prior to beginning formal assessments, the researchers individually studied the biological characteristics, ecological requirements, and care standards of the target species, sharing their knowledge with the team to ensure a common understanding (19, 31, 33). During the assessments, they used various models of mobile phones to take photos and videos of the animals and enclosures, which were then shared within the team to enhance objectivity and minimize subjective interpretations (22, 32, 34). At one large zoo (Table 1, A1), an animal welfare expert with 11 years of experience participated in the assessments, further enhancing the accuracy and depth of the evaluations. At another large zoo (Table 1, A2), two researchers, an animal welfare expert, and a zookeeper with 30 years of experience collaborated to strengthen the reliability of the assessments (23, 27, 35). At one private zoo (Table 1, C3), all three researchers worked together, while at the remaining six private zoos, they conducted individual assessments following standardized procedures and criteria. Despite efforts to standardize and maintain consistency, assessments were conducted by different observers on different dates and at different times due to variations in zoo operating hours, accessibility, and the availability of researchers. After completing their fieldwork, the team reviewed the observations and finalized their assessments through discussions to ensure objectivity and consistency (22, 36, 37).

**Table 1:** Classification of the nine assessed zoos in South Korea.

Zoo grade		Zoos	No. of species	No. of animals	Operation classification	Type (indoor/outdoor)
A	AZA-accredited zoos	A1	237	2,112	public	Mixed
		A2	127	1,543	private	Mixed
B	Unaccredited large zoos with >50 species	B1	50	266	private	indoor
		B2	60	2,897	private	indoor
		B3	79	821	private	Mixed
C	Unaccredited small zoos with <50 species	C1	9	49	private	indoor
		C2	17	694	private	indoor
		C3	41	1,023	private	indoor
		C4	48	298	private	indoor

This table categorizes the nine zoos based on accreditation status, number of species, population, operational classification (public/private), and type (indoor/outdoor). Group A consists of AZA-accredited zoos with a larger number of species and animals. Groups B and C include unaccredited zoos, with Group B housing more than 50 species and Group C fewer than 50 species. Understanding these classifications helps us see how accreditation and zoo size may influence animal welfare outcomes.

### 2.2 Welfare-assessed zoos

As of December 2022, there were 114 registered zoos in South Korea.^19^ To ensure the representativeness and efficiency of the welfare evaluations, nine zoos were selected based on international accreditation status, number of species, and accessibility (Table 1) (8, 30, 38). This selection process aimed to include zoos representing a range of sizes and operational models within South Korea. Group A consisted of two zoos accredited by AZA (See footnote 6) or EAZA; Group B included three private zoos housing over 50 species (See footnote 7); and Group C comprised four private zoos with fewer than 50 species.

### 2.3 Animals

A total of 304 animals from 11 species commonly found in the selected zoos were evaluated. These species were chosen based on the study’s objectives and the care conditions at each zoo to ensure consistency while allowing for the assessment of a diverse range of animals (29, 39, 40). The species included 24 scarlet macaws (*Ara macao*), 27 raccoons (*Procyon lotor*), 62 meerkats (*Suricata suricatta*), 25 fennec foxes (*Vulpes zerda*), 37 sulcata tortoises (*Centrochelys sulcata*), 12 alpacas (*Lama pacos*), 16 capybaras (*Hydrochoerus hydrochaeris*), 21 coatis (*Nasua nasua*), 10 corn snakes (*Pantherophis guttatus*), 54 rabbits (*Oryctolagus cuniculus*), and 16 toco toucans (*Ramphastos toco*).

In cases where the target species were not present, similar species with comparable ecological requirements and care needs were evaluated to maintain the study’s accuracy and representativeness (30, 35, 40). For example, at one large zoo and two private zoos, Amazon parrots (*Amazona* spp.), African gray parrots (*Psittacus erithacus*), and sun conures (*Aratinga solstitialis*) were assessed instead of scarlet macaws due to their similar behavioral characteristics and environmental needs. Similarly, Aldabra tortoises (*Aldabrachelys gigantea*), leopard tortoises (*Stigmochelys pardalis*), red-footed tortoises (*Chelonoidis carbonarius*), and Hermann’s tortoises (*Testudo hermanni*) were evaluated in place of sulcata tortoises at one large zoo and one private zoo, as they share comparable habitat preferences and care requirements. Additionally, Burmese pythons (*Python bivittatus*) and ball pythons (*Python regius*) were assessed instead of corn snakes in one large zoo and two private zoos, given their similar husbandry needs.

### 2.4 Ethical considerations

All assessments were observational and non-invasive, conducted without any direct interaction with the animals to ensure their well-being was not compromised. Ethical approval from Kyungpook National University’s ethics committee was not required, as the study involved non-invasive observations. Permissions from zoo authorities were obtained for Group A zoos, while observations at other zoos were conducted without the need for additional permits, adhering to relevant national guidelines for animal welfare.

### 2.5 Development and structure of S-AWAG

#### 2.5.1 Conceptual development of S-AWAG

S-AWAG is an assessment tool developed by integrating the strengths of both the Animal Welfare Assessment Grid (AWAG), created by Justice et al. (17), and the Modified Animal Welfare Assessment Grid (M-AWAG), developed by Ma et al. (38), while addressing their respective limitations. AWAG provides a foundational framework for animal welfare assessments, evolving into a more systematic, specific, and practical tool based on the Five Domains Model (17, 41, 42). The Five Domains Model categorizes animal welfare into five key domains: ‘Nutrition,’ ‘Environment,’ ‘Health,’ ‘Behavior,’ and ‘Mental State,’ facilitating a comprehensive evaluation of welfare conditions across these domains (43–45). One of the primary strengths of this model is that it considers not only the animal’s physical state but also environmental conditions, behavioral needs, and subjective emotional experiences, enabling a balanced and holistic analysis of animal welfare and ensuring reliable assessments (5, 36, 46). AWAG further minimizes subjective judgment by quantifying welfare conditions into objective scores, dividing each domain into categories of ‘Physical,’ ‘Psychological,’ ‘Environmental,’ and ‘Procedural’ factors to allow for a more detailed assessment of the variables that impact animal welfare (17, 41, 46). Due to this structured approach, AWAG has become a widely adopted standard for animal welfare assessments in various countries (18, 41, 45). S-AWAG builds upon this reliability by incorporating AWAG’s framework and assessment scales.

However, AWAG’s complex design and expert-centered approach make assessments time-consuming and difficult for untrained assessors to use effectively (18, 38, 45). To address these challenges, M-AWAG was developed, adapting AWAG to reflect the unique characteristics of zoos in South Korea. M-AWAG simplifies the assessment items and employs a Likert scale, making evaluations more intuitive and efficient (38). As a result, it boasts high inter-rater reliability (IRR = 0.942) and allows for faster assessments (38, 47). S-AWAG integrates these strengths from M-AWAG, further simplifying the assessment process while maintaining objectivity and consistency. Like its predecessors, S-AWAG offers a structured format for recording assessment results, supporting a systematic approach to welfare improvement (17, 38, 41). While S-AWAG does not include specific features for long-term monitoring, the structured documentation and analysis of results facilitate ongoing monitoring (32, 34, 46). This format also helps assessors focus on clear criteria, enhancing consistency and ensuring reliable assessments across various environments and species (18, 41, 48).

#### 2.5.2 Streamlining the S-AWAG assessment process

The S-AWAG framework is built upon the four key parameters originally proposed in the AWAG system, with each item rated on a scale from 1 (best condition) to 10 (worst condition), similar to the AWAG model (17). However, S-AWAG has been designed for greater accessibility to non-experts and therefore excludes some elements from the original framework, simplifying the assessment to focus on key parameters. Specifically, 11 of the more complex and specialized assessment items from the AWAG system have been removed, and the assessment categories have been streamlined into two main areas: ‘Health’ and ‘Environment.’

In the ‘Health’ section of S-AWAG, all physical factors from the original AWAG have been retained, along with key psychological indicators such as ‘abnormal behavior,’ while other factors were excluded. The ‘Environment’ section has been simplified to include all existing environmental factors except for ‘nutrition,’ focusing mainly on the procedural element of ‘visitors.’ For example, in the ‘Physical’ domain, detailed physiological measurements like blood tests and temperature checks were omitted. In the ‘Psychological’ domain, indicators such as stress hormone levels caused by capture or training, complex behavioral enrichment program analyses, and social behavior assessments were excluded, leaving only the ‘abnormal behavior’ factor. Similarly, in the ‘Environment’ section, detailed nutritional analyses were removed, and in the ‘Procedural’ section, professional items such as ‘veterinary procedures,’ ‘sedation,’ and ‘restraint’ were omitted. These items were replaced with more practical and observable indicators. Despite these simplifications, S-AWAG remains grounded in the core welfare principles of the Five Domains Model, aiming to comprehensively assess both the physical and mental well-being of animals while still capturing the essential welfare indicators (43, 49, 50). Research by Whitham et al. (35) and Meagher (51) has shown that even when welfare assessments rely solely on observable indicators, they can still serve as reliable tools for evaluating welfare. These studies provide strong support for the streamlined approach used in S-AWAG.

#### 2.5.3 Framework and components of S-AWAG

**Figure 1:**
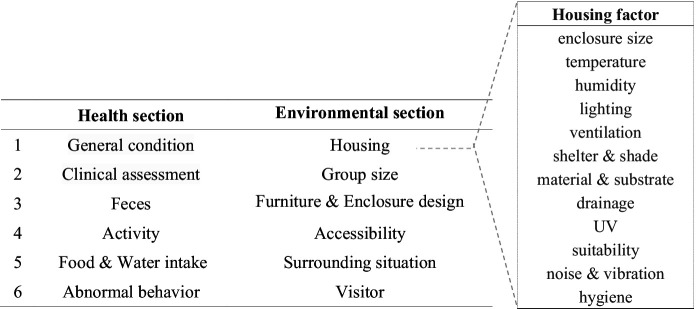
Simplified Animal Welfare Assessment Grid (S-AWAG) structure. This figure illustrates the structure of the S-AWAG, which is composed of two main sections: the ‘Health’ section and the ‘Environmental’ section. The ‘Health’ section provides a comprehensive evaluation of the animals’ physical and psychological well-being, while the ‘Environmental’ section addresses key factors related to the animals’ living conditions. S-AWAG is designed to be easy to use, even by non-experts, providing consistent welfare assessments across zoos of different sizes and conditions. The clear separation between health and environmental factors enables a more comprehensive understanding of animal welfare in different settings.

S-AWAG is composed of two primary sections: the ‘Health’ section and the ‘Environment’ section, designed to evaluate the balance between an animal’s physical and psychological condition alongside its living environment (Figure 1 and Supplementary Table 1).

The ‘Health’ section assesses the animal’s overall physical and psychological state through six factors: ‘general condition,’ ‘clinical assessment’ (visible diseases and injuries), ‘feces’ (consistency of excretions), ‘activity,’ ‘food & water intake,’ and ‘abnormal behavior’ (including stereotypical behaviors). For example, in the ‘general condition’ factor, an animal maintaining its body weight within a normal range (±5% of average weight) receives a score of 1, whereas an animal whose weight is 30% above or below the normal range, or whose coat or feathers are severely damaged, receives a score of 10. The ‘clinical assessment’ factor evaluates the severity of illness or injury, while the ‘feces’ factor indirectly assesses health by examining the condition of the animal’s excretions. The ‘activity’ factor evaluates mobility and physical activity, while the ‘food & water intake’ factor assesses whether the animal is consuming food and water normally, as well as signs of dehydration, hunger, or food refusal. Lastly, in the ‘abnormal behavior’ factor, a score of 1 is given if no stereotypical behaviors (such as pacing or self-harm) are observed, while a score of 10 is given if such behaviors are frequent and severe.

The ‘Environment’ section evaluates the animal’s living conditions through six factors: ‘housing condition,’ ‘group size,’ ‘furniture & enclosure design,’ ‘accessibility,’ ‘surrounding situation,’ and ‘visitors.’ The ‘housing’ factor is further divided into 12 subcategories that assess the suitability of the animal’s living environment. For instance, the ‘enclosure size’ subcategory evaluates whether the space provided meets the individual, behavioral, and social needs of the species. Factors like ‘temperature,’ ‘humidity,’ ‘lighting,’ and ‘ventilation’ assess whether the environmental conditions meet the species’ comfort requirements. The ‘shelter & shade’ subcategory evaluates whether sufficient shelter and shade are available, while the ‘material & substrate’ subcategory assesses the quality of materials and substrates used in the enclosure. The ‘drainage’ subcategory evaluates whether proper drainage is maintained, and the ‘UV’ subcategory assesses whether adequate UV A and B lighting is provided. For instance, a score of 1 is given if both natural light and UV A/B lighting are adequately available. If UV B lighting is only available in certain areas, limiting access for some animals, a higher score is assigned. If no UV lighting is provided, leading to health issues such as rickets, osteoporosis, or appetite loss, the score is 10. The ‘suitability’ factor evaluates the degree of exposure to visitors or other animals. A score of 1 is given if the animal is minimally exposed and experiences little stress, while higher scores are assigned based on the level of exposure, with a score of 10 given if the animal is completely exposed in all directions without any shelter. The ‘noise & vibration’ factor assesses the presence of excessive noise and vibrations in the animal’s environment, originating from visitors or equipment installations/construction activities, while the ‘hygiene’ factor evaluates the cleanliness of the living space. In the ‘group size’ factor, higher scores are assigned as group sizes deviate from natural conditions; severely overcrowded conditions or solitary confinement of social animals result in higher scores. The ‘furniture & enclosure design’ factor examines whether adequate shelter, hiding spaces, nest boxes, branches, and plants are provided to allow animals to express natural behaviors. The ‘accessibility’ factor assesses whether animals can freely move between exhibit areas and holding spaces. Lastly, the ‘surrounding situation’ factor evaluates external disturbances such as enclosure modifications, construction work, or the introduction of new animals, which may affect the animals’ routines and comfort. The ‘visitors’ factor examines the impact of visitors on the animals, considering their presence, number, frequency, and interactions. Scoring is based on the number of visitors within 1 h: a score of 1 is given if there are no visitors; fewer than 50 visitors correspond to a score of 3; 50 to 100 visitors correspond to a score of 5 or 7 (with a score of 7 assigned if negative interactions increase); more than 100 visitors correspond to a score of 9 or 10 (with a score of 10 assigned if negative interactions increase).

This comprehensive approach in S-AWAG enables a thorough and consistent assessment of the welfare conditions experienced by animals, balancing their health and environmental factors to provide a holistic evaluation of animal welfare.

### 2.6 Research method

In this study, the Simplified Animal Welfare Assessment Grid (S-AWAG) was used to assess the welfare status of 11 species across nine zoos in South Korea. We consistently applied the observation-based S-AWAG grid in each zoo to evaluate the animals’ physical and psychological conditions, as well as the environmental conditions of their enclosures (see Section 2.4.3). These assessments provided baseline data for comparing welfare conditions across different zoos. By adopting an observer-based approach, we minimized subjective interpretations and focused on measurable, objective factors, ensuring consistency in our evaluations (35, 51). This method, centered on the physical condition of the animals and their habitats, is crucial for reducing individual observer bias and maintaining objectivity in welfare assessments.

#### 2.6.1 Descriptive statistics

To analyze and interpret the S-AWAG data, SPSS statistical software was utilized (15, 30, 52). The mean and interquartile range (IQR) were calculated to understand the central tendency and variability of the welfare scores. The mean provides an average score, offering a general idea of the overall welfare conditions within each zoo group. The IQR shows the middle 50% of the data, highlighting how much the scores vary within each group. This combination of statistics allowed for the comparison of welfare scores across different zoo groups and the assessment of how consistently welfare standards were maintained. Boxplots were also used to visually represent the ‘Health’ and ‘Environment’ scores across zoo groups. Boxplots are helpful tools that display key statistical information in a simple graphical format. They clearly depicted central tendencies (horizontal lines), variability (IQR), outliers (individual points), and minimum/maximum values (whiskers), making it easier to compare groups at a glance (16, 38, 53).

#### 2.6.2 Statistical differences in S-AWAG scores by zoo group

To determine whether there were significant differences in welfare scores among the zoo groups, the zoos were categorized into three groups (A, B, C) based on their international accreditation and the number of species housed (Table 1). A one-way analysis of variance (ANOVA) was performed using SPSS software (30, 38, 52). ANOVA is a statistical technique used to compare the means of three or more groups to see if there are any statistically significant differences among them. In this study, ANOVA helped identify whether the differences in welfare scores between Groups A, B, and C were meaningful or simply due to random chance. After finding significant differences, Tukey’s post-hoc test was conducted, and Hochberg’s *p*-value correction was applied to adjust for multiple comparisons and enhance the reliability of the results (33, 52, 54). The significance level (*α*) was set at 0.05, meaning that differences with a p-value less than 0.05 were considered statistically significant (52, 54). This approach allowed for confidently identifying meaningful differences in welfare scores among the zoo groups.

#### 2.6.3 Inter-rater reliability of S-AWAG

Inter-rater reliability (IRR) measures how consistently different assessors evaluate the same subjects. To evaluate the consistency among the three researchers who conducted the welfare assessments, Fleiss’s kappa and Cohen’s kappa were calculated using SPSS software (37, 47, 52). Fleiss’s kappa is suitable for assessing agreement among three or more raters, while Cohen’s kappa is used for pairs of raters. Linear weighting was applied to account for differences in the severity of ratings between observers, providing a more nuanced analysis of agreement. An IRR value (kappa) of 0.8 or higher is generally considered to indicate very high agreement among assessors, ensuring the reliability of the assessment tool (28, 31, 47). High IRR values in this study suggest that the S-AWAG produces consistent results regardless of who uses it, which is particularly important when non-experts are involved in the assessment process.

#### 2.6.4 Methodology for Pearson correlation analysis of health and environmental factors

To explore the relationships between the ‘Health’ and ‘Environment’ sections of the S-AWAG, Pearson correlation coefficient (PCC) analysis was performed using SPSS software (28, 52, 53). The Pearson correlation coefficient is a statistical measure that indicates the strength and direction of a linear relationship between two variables. This method was chosen because it is intuitive and accessible, even to non-experts, making it easier to interpret the relationships between variables. Values close to 1 indicate a strong positive correlation, meaning that as one variable increases, the other also increases. Values close to −1 indicate a strong negative correlation, where one variable increases as the other decreases. Values around 0 suggest little to no linear relationship. Generally, a PCC value of 0.5 or higher (or −0.5 or lower) indicates a moderate to strong correlation, suggesting meaningful associations between variables (52). By identifying these relationships, it became possible to understand how different aspects of animal welfare interact with each other. This information is valuable for prioritizing areas for improvement and developing more effective welfare strategies.

## 3 Results

The welfare assessment conducted using S-AWAG across nine zoos in South Korea revealed significant disparities in animal welfare standards based on zoo group classification, accreditation status, and management practices (Table 1). These differences were evaluated through various statistical methods to ensure robustness and reliability.

**Figure 2:**
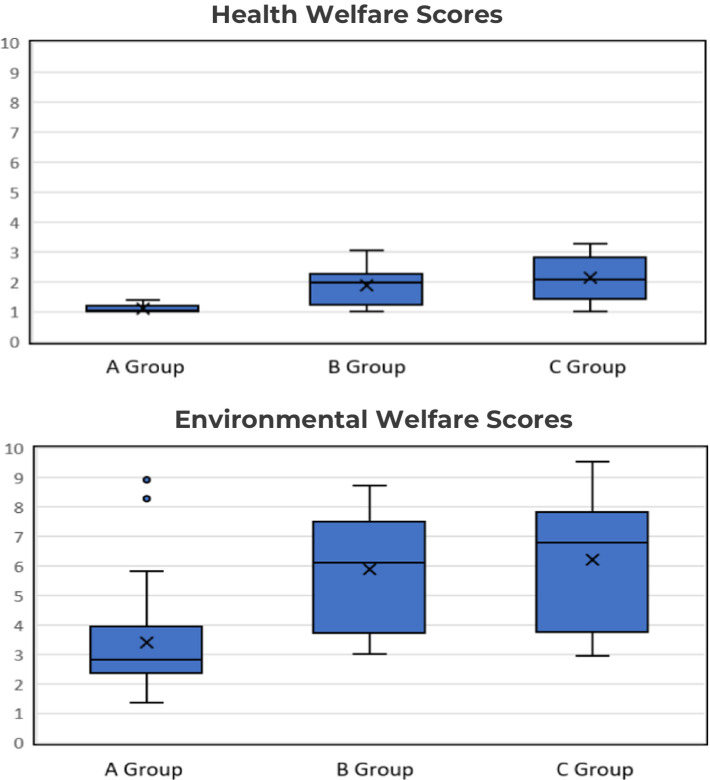
Box plot of S-AWAG scores for 11 animal species across zoo groups. The *y*-axis shows S-AWAG scores (1 = best, 10 = worst), and the *x*-axis categorizes zoo groups (Group A: AZA-accredited zoos, Group B: large unaccredited zoos, and Group C: small unaccredited zoos). The boxplots display the median, interquartile range (IQR), and any outliers. The central line in each box indicates the median (50th percentile), and the box encompasses the interquartile range (IQR) between the 25th and 75th percentiles. The whiskers extend to 1.5 times the IQR, highlighting the range of the data, while outliers are shown as individual points beyond the whiskers. In the health welfare plot, Group A has the narrowest range and lowest median, suggesting better welfare conditions, while Groups B and C show greater variability. Similarly, in the environmental welfare plot, Group A displays lower scores, whereas Groups B and C show higher variability and poorer welfare. These boxplots highlight welfare disparities across zoo groups, particularly emphasizing areas for improvement, particularly in smaller, non-accredited zoos.

The average score in the ‘Health’ section across all zoos was 1.65, whereas the average score in the ‘Environment’ section was significantly higher at 5.51. This stark contrast highlights that while zoos performed relatively well in maintaining the health of the animals, they struggled to provide appropriate environmental conditions. As illustrated in Table 2 and Figure 2, significant differences in welfare levels were evident between the zoo groups, indicating that the number of species and accreditation status have a notable impact on welfare conditions. Group A achieved the best scores, with 1.09 in the ‘Health’ section and 3.48 in the ‘Environment’ section, indicating consistent welfare standards as evidenced by smaller interquartile ranges (IQRs) and lower medians in the boxplots. Despite the presence of outliers in Group A—suggesting that some animals experienced poorer-than-average welfare conditions—the group still showed significantly better standards than Groups B and C. Group B had a higher ‘Health’ score (1.87) and recorded 5.77 in the ‘Environment’ section, which was better than Group C but still lagged behind Group A. Group C exhibited a wider score distribution, with a ‘Health’ score of 1.76 and the highest ‘Environment’ score of 6.33, reflecting poorer and more inconsistent welfare conditions. These results suggest that non-accredited zoos, particularly smaller ones, face greater challenges in maintaining consistent welfare standards.

**Table 2:** Average S-AWAG scores for health & environmental sections across zoo groups.

	Group A	Group B	Group C
Health section	1.09	1.87	1.76
Environmental section	3.48	5.77	6.33
Means	2.28	3.82	4.04

This table shows the average welfare scores for the ‘Health’ and ‘Environment’ sections of the S-AWAG across the three zoo groups (*α* = 0.05) Lower scores indicate better welfare conditions. Group A (AZA-accredited zoos) has the lowest average scores, suggesting better overall welfare. Groups B and C (unaccredited zoos) have higher scores, indicating poorer welfare conditions. Notably, Group C has the highest average environmental score, highlighting challenges faced by smaller, unaccredited zoos in providing adequate environments for the animals.

The ANOVA results presented in Table 3 clearly highlight significant differences in welfare levels among the three groups. Statistically significant differences were found in both the ‘Health’ and ‘Environment’ sections between Groups A, B, and C. Notably, the *p*-value for the ‘Environment’ section was less than 0.001, indicating very strong statistical significance and suggesting that the environmental differences among the groups are the result of actual management practices rather than random variation (52, 54). Similarly, the *p*-value for the ‘Health’ section was below 0.05, confirming that the differences between the groups were meaningful and not due to chance. Further analysis using Tukey’s post-hoc test and Hochberg’s *p*-value correction provided additional insights (52). Tukey’s test revealed that Group A consistently outperformed Group C, especially in environmental conditions, reflecting the benefits of accreditation and better resources. Group B performed better than Group C but still lagged behind Group A. Hochberg’s correction ensured the robustness of these findings, confirming that the differences, particularly between Group A and Group C, were statistically significant.

**Table 3:** ANOVA results for health and environmental scores among zoo groups.

Health section	Environmental section
A < B < C (*p*-value<0.05)	A < B < C (*p*-value = 0.00)

The ANOVA results indicate that the differences in welfare scores among the zoo groups are statistically significant. Specifically, the order A < B < C shows that Group A (accredited zoos) has the best welfare scores, followed by Group B, and then Group C. ANOVA assesses whether these observed differences are meaningful or could have occurred by chance. Although Group C may have a lower average score than Group B, the difference might not be statistically significant, whereas the difference between Groups A and B is more substantial, leading to the conclusion of A < B < C. The significance level (*α* = 0.05) represents a 5% risk of incorrectly identifying a difference where none exists. In both the ‘Health’ and ‘Environment’ sections, *p*-values below 0.05 confirm that the differences between the zoo groups are statistically significant and unlikely due to random chance. Notably, the *p*-value of 0.00 in the ‘Environmental’ section strongly suggests that the differences in environmental conditions are due to actual management practices rather than random variation. These results highlight the critical role of accreditation and effective zoo management in maintaining higher welfare standards.

The inter-rater reliability (IRR) analysis, as shown in Table 4, demonstrated high levels of agreement among the three researchers who conducted the assessments. The IRR between individual pairs of researchers exceeded 0.930 (*A*&*B*: 0.930, *A*&*C*: 0.951, *B*&*C*: 0.932), indicating that the S-AWAG tool allows for consistent and objective assessments even across different observers (47, 52). Although the overall IRR was slightly lower at 0.839, it still supports the robustness and reliability of the assessment tool, affirming that non-experts can consistently apply the welfare criteria with minimal variation in their assessments (47, 52).

**Table 4:** Inter-rater reliability (IRR) analysis between researchers for S-AWAG assessment.

Researchers	A&B	A&C	B&C	All researchers
Inter-rater reliability	0.930	0.951	0.932	0.839

This table presents the agreement levels among the three researchers who conducted the assessments. We measured their scores using Cohen’s kappa for each pair of researchers and Fleiss’s kappa for all three. The inter-rater reliability (IRR) values are all above 0.8, indicating a very high level of agreement among the evaluators. Since kappa values of 0.8 or higher generally signify strong consistency, these results enhance the credibility of the S-AWAG tool. The *p*-values of 0.000 in all cases confirm that the observed agreement is statistically significant (*α* = 0.05), meaning the consistency among evaluators is unlikely due to chance. This demonstrates the tool’s robustness and objectivity in assessing animal welfare.

**Figure 3:**
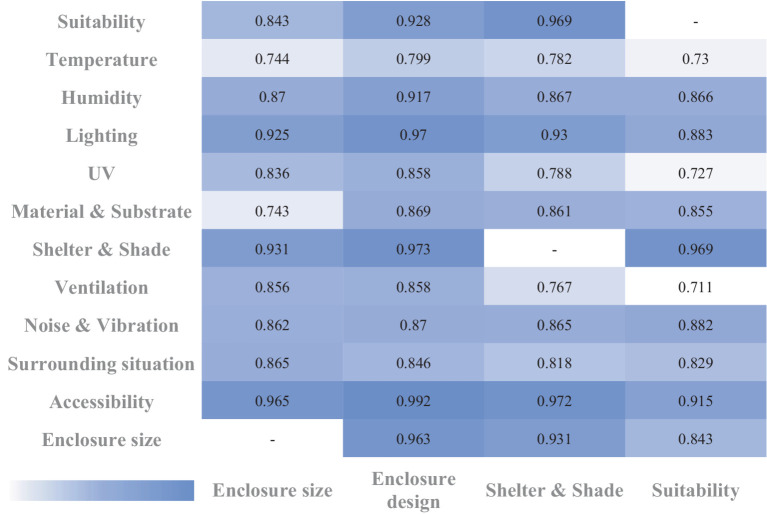
Pearson correlation coefficients for environmental sector factors (*α* = 0.05). This heatmap illustrates the Pearson correlation coefficients (PCC) between different environmental factors. The PCC measures the strength and direction of the linear relationship between two variables, with values close to 1 indicating a strong positive correlation and values close to −1 indicating a strong negative correlation. Generally, a PCC of 0.5 or higher (or −0.5 or lower) suggests a moderate to strong correlation, indicating meaningful associations between variables. Each square in the heatmap reflects the strength of the correlation, with darker shades representing stronger relationships. We set the significance level at *α* = 0.05; thus, correlations with *p*-values below this threshold are considered statistically significant, reducing the likelihood that the observed correlations are due to random chance. This visualization underscores the importance of environmental factors in influencing animal welfare, highlighting areas that zoos should prioritize for welfare enhancement.

**Figure 4:**
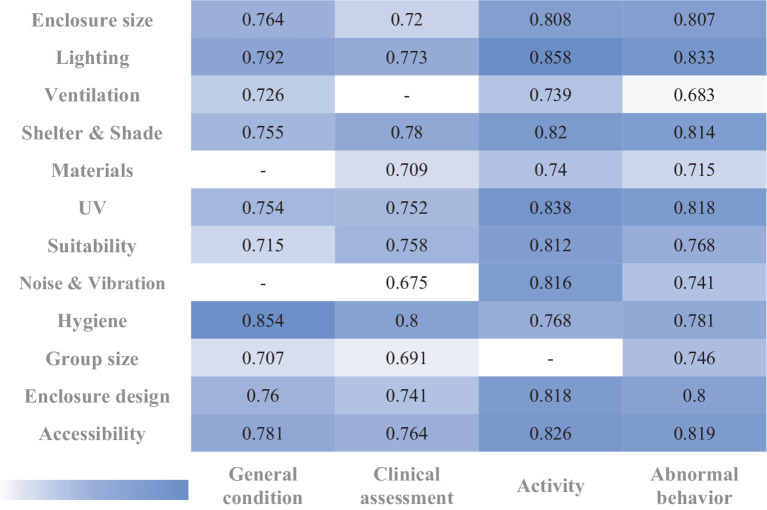
Pearson correlation coefficients between health & environmental factors (*α* = 0.05). This heatmap illustrates the Pearson correlation coefficients (PCC) between health and environmental factors. The PCC measures the strength and direction of the linear relationship between two variables, with values close to 1 indicating a strong positive correlation and values close to −1 indicating a strong negative correlation. Generally, a PCC of 0.5 or higher (or −0.5 or lower) suggests a moderate to strong correlation, indicating meaningful associations between variables. Each square in the heatmap reflects the strength of the correlation, with darker shades representing stronger relationships. We set the significance level at *α* = 0.05; thus, correlations with *p*-values below this threshold are considered statistically significant, reducing the likelihood that the observed correlations are due to random chance. This visualization underscores the importance of environmental factors in influencing animal welfare, highlighting areas that zoos should prioritize for welfare enhancement.

Pearson correlation coefficient (PCC) analysis revealed strong correlations both within and between the ‘Health’ and ‘Environment’ sections. For instance, as shown in Table 5, there was a strong correlation between ‘clinical assessment’ and ‘abnormal behavior’ (*PCC =* 0.984) and between ‘activity’ and ‘abnormal behavior’ (*PCC =* 0.957), demonstrating that as health deteriorates, abnormal behavior increases and activity decreases. Figure 3 illustrates that the highest correlation was between ‘shelter & shade’ and ‘furniture & enclosure design’ (*PCC =* 0.973), underscoring the importance of adequate shelter and shade for animal welfare. Additionally, the strong correlation between ‘enclosure size’ and ‘furniture & enclosure design’ (*PCC =* 0.963) suggests that even in large enclosures, poor design can negatively affect welfare, emphasizing the importance of the quality of the enclosure environment beyond mere physical space (1, 17, 43). Moreover, the correlation between ‘ventilation’ and ‘furniture & enclosure design’ (*PCC =* 0.858) underscores the necessity of good ventilation for maintaining animal health. Figure 4 shows the correlation between ‘enclosure size’ and ‘abnormal behavior’ (*PCC =* 0.807), indicating that restricted space is likely to cause stress and abnormal behaviors in animals. The correlations between ‘hygiene’ and health maintenance were also very high, ranging from 0.768 to 0.854, emphasizing the critical role of a clean environment in maintaining overall animal health and preventing diseases. The strong correlation between ‘accessibility’ and ‘activity’ (*PCC =* 0.826) is noteworthy, indicating that free movement between the exhibit and holding areas is crucial for animal welfare.

**Table 5:** Pearson correlation coefficients for health sector factors (*α* = 0.01).

	General condition	Clinical assessment
Activity	0.934	0.957
Abnormal behavior	0.947	0.984

This table presents the Pearson correlation coefficients between key health factors in the S-AWAG assessments. All correlations are significant at the 0.01 level (*α* = 0.01). The strong correlations indicate the close relationships among various health factors, highlighting the influence of one health indicator on others. Understanding these relationships helps in developing comprehensive strategies to improve animal health and welfare.

**Figure 5:**
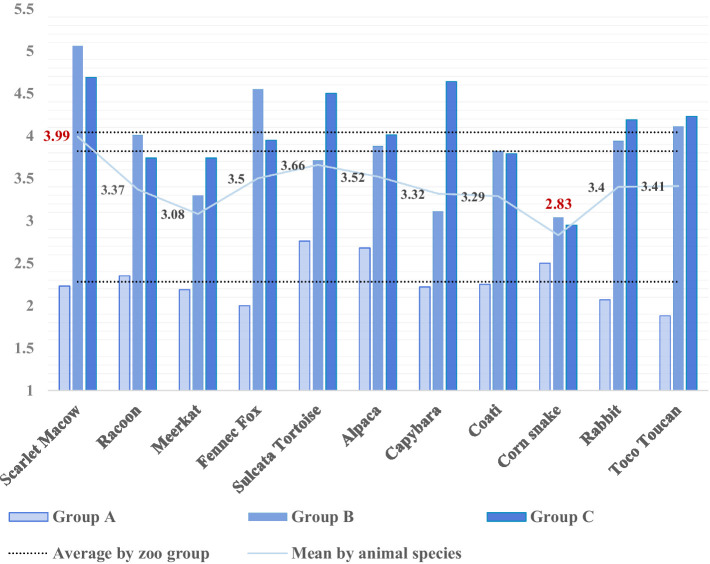
Welfare score comparison of 11 animal species across zoo groups. This figure compares welfare scores (*y*-axis: 1 = best, 5 = worst) for 11 animal species across three zoo groups (Group A: AZA-accredited zoos, Group B: large non-accredited zoos, and Group C: small non-accredited zoos). Each bar represents the average welfare score for a specific animal species within the zoo group, and the light blue line connects the overall average scores for each species, facilitating cross-species comparisons. Group A consistently shows lower scores, indicating better welfare conditions, while Group C shows higher scores, pointing to poorer welfare. This comparison highlights species-specific welfare conditions and underscores disparities between zoo groups, guiding targeted welfare improvements.

Figure 5 visually compares the welfare scores of 11 animal species across the three zoo groups. Group A exhibited relatively good welfare conditions, with most animals scoring between 2.0 and 2.5, indicating better welfare. In contrast, Groups B and C recorded scores above 3.5, indicating poorer welfare conditions. Group C, in particular, had the highest scores across most species, suggesting that small non-accredited zoos struggle to maintain welfare standards due to limited physical space and a lack of environmental enrichment.

Among the species evaluated, the scarlet macaw received the highest average score (3.99), reflecting the challenging environment in which they live, particularly in Group B. Conversely, the species with the lowest (best) average score was the corn snake, with a score of 2.83, and it also displayed the smallest gap between groups. These findings highlight species-specific welfare issues that require targeted interventions.

## 4 Discussion

### 4.1 The originality and accessibility of S-AWAG: an effective welfare assessment tool for non-experts

The Simplified Animal Welfare Assessment Grid (S-AWAG) stands out due to its simple and intuitive design, making it easily accessible to non-experts. This accessibility offers several key advantages over existing welfare assessment tools, which can be summarized in three main points:

First, S-AWAG is designed for ease of use by individuals without specialized training. Existing tools often require complex procedures and a high level of expertise, focusing on specialized approaches tailored to specific species or environments (18, 26, 45). While such tools may be effective in large public zoos or research-focused settings, they are difficult to apply in small-scale zoos where non-experts are more commonly involved (18, 23, 55). In contrast, S-AWAG provides specific and clear evaluation items, allowing assessors to focus on key welfare indicators while minimizing subjective judgments and emphasizing observable factors (see Section 2.5.3) (17, 41, 45). This design reduces confusion and errors during the assessment process, enabling non-experts to conduct consistent evaluations without the need for complex knowledge (22, 28, 31). Moreover, S-AWAG incorporates straightforward statistical methods, such as Pearson correlation analysis, which can be easily applied using standard statistical software without requiring extensive mathematical modeling or advanced statistical expertise (22, 28).

Second, S-AWAG is designed for speed and efficiency. In small non-accredited zoos, where resources and manpower are often limited and where poor welfare conditions urgently require improvement, it is crucial to perform objective, data-driven assessments quickly (14–16). To achieve this, S-AWAG incorporates simplified and clear evaluation indicators and a streamlined statistical system, which shortens the data collection and analysis process, allowing for rapid gathering of essential information (28, 51, 53). This ensures that even in resource-constrained facilities, effective assessments can be conducted with minimal training and guidance, enabling quick evaluations and immediate responses (15, 31, 52). Additionally, this swift evaluation process aids in the early detection and resolution of welfare issues before they escalate, preventing more severe conditions (36, 46, 49). Furthermore, the simplified process of selecting assessors allows for rapid deployment in various environments, which is another key advantage of S-AWAG (31, 50).

Third, S-AWAG adopts a non-invasive approach, enhancing both practicality and safety. The tool is designed to minimize disturbance to animals, allowing them to maintain their natural behavior during welfare assessments (1, 18, 49). This non-invasive method facilitates repeated assessments over time without causing long-term harm or distress, promoting continuous monitoring and timely interventions (35, 48, 51). By minimizing the need for invasive procedures, S-AWAG also reduces the risk of zoonotic disease transmission, making it safer and more practical for frequent use (19, 32, 43).

### 4.2 S-AWAG: a comprehensive tool for consistent welfare assessments across diverse contexts

Originally developed by Justice et al., the Animal Welfare Assessment Grid (AWAG) has been used to assess a range of animals, including primates and birds, and has since been adapted to a wide variety of species—from farm animals (34) and dogs (53) (*Canis lupus familiaris*) to large felids (41), giraffes (32) and marine animals, including decapods, cephalopods, and even large mammals like whales (42, 46). Similarly, in this study, S-AWAG was able to statistically distinguish between welfare levels at large, accredited zoos and smaller, non-accredited zoos (*p* < 0.05, *p* = 0.00), confirming its applicability across multiple species (11 species in this study) (Table 3 and Figure 5). This demonstrates that S-AWAG, based on AWAG, is an effective and reliable tool for comparing and evaluating welfare levels in diverse environments. Moreover, it systematically accounts for the specific environmental needs of each species, ensuring consistent assessments. However, as outlined in the study’s procedures, pre-training and prior research are necessary to ensure the consistent use of S-AWAG. Adequate training helps assessors accurately understand the tool, and prior research on the target animals and their environments minimizes variables, thereby improving the consistency and reliability of the assessments (4, 18, 19).

S-AWAG is also designed to allow users from various backgrounds to conduct consistent welfare assessments. Studies by Malkani et al. (53) and Brouwers and Duchateau (28) reported very high inter-rater reliability (IRR) of 0.97 and 0.92, respectively, at *p* < 0.001 in AWAG-based experiments. Additionally, Bacon et al. (19) and Webb et al. (31) demonstrated that zoo managers and non-experts from different were able to conduct reliable welfare assessments using the tool. Similarly, in this study, high inter-rater reliability (IRR) and agreement among researchers (Table 4) indicate that even when assessors from diverse backgrounds use S-AWAG, the results remain consistent. This confirms that S-AWAG is a reliable tool for welfare assessments and demonstrates that individuals from varying backgrounds can successfully conduct consistent evaluations across different environments and species (37, 47, 52).

### 4.3 Various analytical methods of S-AWAG

Compared to existing welfare assessment tools, S-AWAG offers a more practical solution for small, non-accredited zoos. Traditional tools often require specialized equipment, extensive training, and significant time investments, which are not feasible for facilities with limited resources (15, 18, 45). One of the primary advantages of S-AWAG is its versatility in offering a range of analytical approaches. These methods not only help identify areas for improvement within welfare assessments but also provide tailored strategies to address the unique needs of individual species. By incorporating the diverse approaches outlined below, S-AWAG significantly enhances animal welfare management, empowering even resource-constrained zoos to achieve meaningful welfare improvements.

#### 4.3.1 Utilizing descriptive statistics to identify welfare improvement areas by group

Descriptive statistics are essential for clearly identifying differences in welfare levels between zoo groups and pinpointing specific areas that require improvement (15, 30, 38). For example, as shown in Figure 2, the welfare scores for the ‘Health’ section in Group A are clustered close to 1, with a narrow score distribution. This indicates that animals in Group A are generally in good welfare conditions, and the zoos in this group are maintaining consistent standards. However, in the ‘Environment’ section, Group A shows somewhat higher scores with a wider distribution, including some outliers. This suggests that even in accredited zoos, certain animals may be in poorer conditions than the overall group, highlighting the need for targeted management specifically for those outliers. In contrast, Table 2 shows that Group B recorded the poorest scores in the ‘Health’ section, while Group C had the highest scores in the ‘Environment’ section. This implies that smaller zoos may face challenges with proper management and securing necessary resources, indicating that Group B and Group C each have distinct areas requiring welfare improvements. Moreover, while Group C performed better than Group B in the ‘Health’ section, the wide score distribution in Figure 2 suggests that some animals in Group C may be in particularly poor conditions. These findings reflect the ongoing challenges that small, non-accredited zoos face in maintaining consistent welfare standards due to regulatory gaps and resource limitations (8, 15, 16). By analyzing descriptive statistics, the welfare levels in each group can be clearly identified, enabling the development of targeted strategies to address outlier species in Group A, enhance health management in Group B, and implement overall welfare improvements in Group C.

#### 4.3.2 Utilizing descriptive statistics to identify species-specific welfare improvement areas

S-AWAG provides a clear indication of which species within each group require more focused welfare improvements. For instance, the scarlet macaw recorded a score of 2.23 in Group A, indicating relatively good welfare, but exhibited a score of 5.06 in Group B, reflecting the worst welfare conditions among all species, and slightly improved to 4.69 in Group C (Figure 5). Similarly, the toco toucan had the lowest score in Group A (1.88), but its scores increased to 4.11 and 4.23 in Groups B and C, respectively. These variations suggest that smaller zoos face challenges in maintaining adequate welfare levels for birds. In this study, we observed that the scarlet macaw housed in Group B was kept alone in a confined space with poorly defined indoor and outdoor areas, inadequate shelter, insufficient lighting, poor ventilation, and a lack of suitable materials and perches. This resulted in self-harm and abnormal behaviors such as feather plucking and lethargy. The presence of scattered feathers on the floor and the overall unhygienic state of the enclosure highlighted poor sanitary conditions. Similar issues were also observed in other species, such as capybaras, rabbits, and fennec foxes. These animals are particularly sensitive to environmental changes, including social interactions, physical activity, and specific temperature and humidity requirements (35, 48, 49). In our observations in Group C, some social animals were kept in isolation, leading to abnormal behaviors like pacing, underscoring the difficulty small zoos face in meeting the ecological, physiological, and social needs of these species.

Conversely, in Group A, which generally showed good welfare conditions, reptiles exhibited notably higher welfare scores compared to other species, suggesting a potential “Criteria Shift Effect,” where animals in generally good condition are evaluated more critically relative to others in the same group. Alternatively, this may reflect that the specific needs of reptiles were not adequately addressed in Group A. Unlike other species, reptiles rely heavily on precise temperature and environmental stimuli rather than physical movement (56, 57). Although Group A zoos typically maintain high welfare standards, they may have lacked the specific environmental adjustments necessary for certain reptile species. To enhance reptile welfare, it is crucial to design enclosures that cater to their particular needs, such as maintaining appropriate temperature and humidity levels, providing adequate heat sources and UVB lighting, and incorporating hiding spots and enclosure designs that replicate their natural habitats to minimize stress (56, 57).

#### 4.3.3 Resource-based welfare improvement strategies tailored to species-specific needs for small zoos

Designing environments that reflect the ecological and physiological needs of animals is essential for enhancing welfare. S-AWAG demonstrates that significant welfare improvements can be achieved with relatively simple management tailored to the unique requirements of each species. For example, the corn snake received a favorable welfare score of 2.83 on average, while the scarlet macaw recorded a relatively poor score of 3.99 (Figure 5). This difference illustrates how species-specific needs are reflected in welfare scores.

Birds like the scarlet macaw require ample flight space, perches, and various environmental stimuli (49, 58, 59). Without these elements, the risk of muscle atrophy, cardiovascular issues, and self-harm increases (55, 56, 59). Therefore, improving their welfare necessitates providing larger spaces and diverse stimuli. In contrast, reptiles like the corn snake have relatively simple environmental needs and do not require the same large spaces as birds for flying (56, 57). As long as temperature and humidity are properly controlled and basic hiding places are provided, they can maintain stable welfare conditions (56, 57). Owing to these straightforward management requirements, the corn snake achieved a good welfare score, demonstrating that welfare can be improved with relatively simple measures for species with less complex needs. This approach contributes to the overall efficiency of welfare programs, allowing managers to allocate more resources and attention to species with complex needs while maintaining good welfare for species with simpler needs using minimal resources (35, 36, 45).

#### 4.3.4 Integration of Pearson correlation analysis and S-AWAG for systematic welfare improvement

The combination of Pearson correlation analysis with the S-AWAG assessment tool plays a critical role in understanding the interactions between various animal welfare factors and establishing systematic strategies for welfare improvement (22, 28, 53). This method helps integrate and analyze key factors, identify weaker correlations that offer valuable insights, and prioritize areas for improvement by comparing the strength of these correlations.

For example, as shown in Table 5, all correlations were significant at the 0.01 level, with several exceeding 0.9, indicating very strong relationships. This analysis suggests that when physical health declines, psychological well-being tends to deteriorate as well, highlighting the importance of using tools like S-AWAG, which comprehensively assess both physical and psychological health to provide more accurate evaluations and drive welfare improvements (35, 41, 43).

Interestingly, the ‘visitor’ factor exhibited weak negative correlations with various welfare indicators, with low statistical significance—particularly with ‘noise & vibration’ and ‘surrounding situation,’ where no significant correlation was observed. In contrast, other ‘Health’ and ‘Environmental’ factors displayed strong positive correlations both within and across sections (Table 5 and Figures 3, 4). This suggests that animals are more sensitive to physical environmental conditions—such as enclosure size, design, and temperature/humidity control—than to the mere presence or frequency of visitors (48, 60, 61). Additionally, it indicates that the psychological and physical states of the animals have a more significant influence on their behavior than visitor presence or number. On the other hand, a strong correlation was found between ‘group size’ and other ‘Health’ factors, yet no significant correlation was observed between ‘Health’ factors and the ‘visitor’ element. This confirms that social species are more influenced by interactions with conspecifics than by non-conspecific interactions, such as those with zoo visitors. This finding aligns with previous studies, which highlight that group dynamics and social enrichment play a more profound role in affecting social animals than interactions with visitors (60, 61). Based on these results, it may be more effective to prioritize direct environmental factors and animal management strategies, rather than focusing on visitor-related policies, when seeking to improve animal welfare.

Furthermore, while ‘enclosure size’ had very high correlations with ‘Environmental’ factors such as ‘enclosure design,’ ‘shelter & shade,’ and ‘materials & substrate,’ it showed relatively lower correlations with ‘Health’ factors (Table 5 and Figure 3). This suggests that simply providing larger enclosures does not necessarily lead to welfare improvements. Without adequate environmental stimulation, even large enclosures can lead to lower activity levels and abnormal behaviors in animals (1, 41, 48). To address this, the design of enclosures, social interaction, and environmental enrichment must be considered alongside enclosure size to improve welfare (40, 44, 55).

Finally, Figure 4 highlights the high correlations between factors like ‘hygiene,’ ‘lighting,’ ‘UV,’ ‘suitability,’ and ‘shelter & shade,’ suggesting that these areas require prioritized improvement. For example, enhancements in ‘hygiene’ and ‘lighting’ showed strong correlations, indicating that these are essential areas to address first. Focusing on these relatively simple factors can lead to significant improvements in animal health and behavior at a low cost. This prioritization allows small-scale zoos, which may have limited resources and staff, to set clear priorities and efficiently improve welfare based on these findings (17, 18, 49).

#### 4.3.5 In-depth evaluations for goal-oriented welfare improvement

By integrating Pearson correlation analysis with species-specific welfare scores across zoo groups, a goal-oriented strategy for improving animal welfare can be effectively developed. This approach facilitates a comprehensive examination of data points, identifying areas that require immediate attention for targeted improvement.

For instance, the Sulcata tortoise in Group A received a relatively high welfare score of 3.36, including a maximum score of 10 in the ‘shelter & shade’ category (Figure 5). This highlights the species’ sensitivity to environmental changes, particularly regarding shelter and shade, emphasizing the need for improvement in these areas (56, 57). Pearson correlation analysis further revealed strong correlations between ‘shelter & shade,’ ‘lighting,’ and ‘UV’ (Figure 3). These findings underscore the importance of maintaining a proper balance between sunlight exposure and adequate shade to enhance the welfare of species like the Sulcata tortoise (56, 57).

Additionally, the scarlet macaw in Group B scored 10 points in both the ‘shelter & shade’ and ‘accessibility’ categories. Analyzing the correlations between these factors yielded several critical insights. First, high correlations were observed between ‘shelter & shade,’ ‘accessibility,’ and other factors such as ‘enclosure size,’ ‘enclosure design,’ ‘materials & substrate,’ ‘activity,’ and ‘abnormal behavior’ (Figures 3, 4). This suggests that providing adequate shelter and shade can significantly improve animal welfare by reducing abnormal behaviors and promoting psychological stability. In particular, the strong correlation between ‘shelter & shade’ and ‘enclosure design’ (*PCC =* 0.973) emphasizes the importance of offering secure spaces where animals feel protected from external stressors. Furthermore, designing enclosures that allow free movement between exhibit and holding areas positively impacts welfare by increasing opportunities for physical activity and interaction with the environment, enhancing both physical and psychological well-being. These findings illustrate the pivotal role that specific environmental factors play in welfare improvement. By focusing on goal-oriented strategies tailored to the needs of particular species, zoos can make significant strides in enhancing animal welfare, regardless of their size or available resources (18, 25, 43).

### 4.4 Limitations and improvement suggestions for S-AWAG

While S-AWAG offers numerous advantages in animal welfare assessments, several critical limitations must be considered.

First, during the simplification process of S-AWAG, essential welfare factors such as ‘enrichment,’ ‘nutrition,’ and ‘social status’—key components in assessing an animal’s overall welfare—may not have been sufficiently incorporated. Excluding or undervaluing these elements in the evaluation could distort or render the assessment results unreliable (17, 35, 38). Additionally, while S-AWAG focuses on quantitative indicators effective for numerically scoring welfare, it may not adequately capture qualitative elements like positive welfare states, emotional well-being, and human-animal interactions. Evaluating these qualitative aspects is crucial because they provide deeper insights into an animal’s subjective experiences and overall satisfaction with its environment (25, 35, 36). For example, indicators such as ‘playfulness,’ ‘curiosity,’ and expressions of natural behavior are vital for assessing how content an animal is within its surroundings (37, 43, 55). Similarly, interactions between animals and humans—whether through gentle handling, enrichment activities, or stressful interactions in crowded viewing conditions—are significant welfare indicators (1, 19, 43). However, such qualitative aspects are difficult to measure quantitatively and may be easily overlooked in assessments, risking the underestimation of an animal’s true welfare level and leading to incomplete evaluations (36, 41, 55). To address these concerns, it is necessary to supplement the assessment with additional items or modules that account for these qualitative factors and capture the full spectrum of animal welfare.

Second, S-AWAG may face issues with representativeness and reliability due to low evaluation frequency and small sample sizes. Infrequent evaluations may not accurately reflect transient states or changes, and relying solely on short-term observational data can limit the assessment’s ability to capture the dynamic nature of animal welfare, including seasonal variations and long-term trends. Factors such as reproductive cycles, social dynamics within groups, and environmental changes can vary over time, making it difficult to capture these elements through a single evaluation (40, 49, 55). Therefore, implementing a long-term monitoring system to continuously track welfare changes is essential (28, 41, 48). Additionally, the small sample size and limited geographic scope of the current evaluations restrict the generalizability of S-AWAG’s results. To address this limitation, further research should be conducted across various zoos and environments to enhance the reliability and expand the applicability of S-AWAG (17, 33, 36).

Third, the reproducibility of assessments using S-AWAG may be challenged by variations in observation times, dates, assessors, and data collection equipment. Due to practical constraints such as zoo operating hours, accessibility, and researcher availability, assessments in this study were conducted at different times of day and on various dates across the nine zoos. Additionally, different data collection tools and equipment, such as cameras and recording devices, were used, potentially affecting the consistency of observations. Different assessors conducted evaluations at different sites, and despite efforts to standardize training and assessment criteria, observer bias or inconsistencies in data collection may have arisen. These factors introduce variability into the data, affecting the reliability and comparability of the results and making it challenging to replicate the study precisely. To improve reproducibility, it is essential to standardize assessment procedures—including consistent observation times, uniform data collection equipment, thorough training of assessors, and clear guidelines for using the S-AWAG tool (17, 18, 26). Implementing protocols for data collection and ensuring that assessors are blinded to certain variables, such as the accreditation status of zoos, can minimize bias and enhance the reliability of the assessments (26, 45). Addressing these methodological considerations in future studies will increase the reproducibility and robustness of welfare assessments using S-AWAG.

Fourth, S-AWAG may not fully account for regional characteristics or the unique needs of specific species. Differences in animal welfare standards can arise from factors such as national laws, animal management practices, and climatic conditions (7, 15, 19). Failure to incorporate these variations into the assessment process could hinder S-AWAG’s international applicability. For example, some countries prioritize certain welfare standards more than others, and regional climate conditions and husbandry practices might necessitate different evaluation criteria. Consequently, customized versions of S-AWAG may be required to reflect the legal standards and practices of each country (15, 30, 38). While S-AWAG can assess a wide variety of species, it may have limitations when evaluating animals with unique physiological and ecological traits, such as marine mammals or large herbivores (42, 46, 50). Assessing these specialized environments or encompassing the diverse needs of various species may require tools tailored to specific species or more comprehensive evaluation frameworks (33, 49, 50). Continuous research and development of these tools will enable more comprehensive welfare evaluations for a wider range of species and environments.

Finally, the limitations of S-AWAG may become more pronounced when non-experts participate in the assessment process. Non-experts may lack the specialized knowledge and experience needed to accurately evaluate animal welfare, potentially resulting in the omission or undervaluation of important welfare factors (23, 26, 27). Consequently, the welfare status of animals may not be accurately reflected, potentially leading to ethical concerns. Furthermore, if non-experts do not fully understand the purpose or functionality of the tool, they may misapply evaluation criteria or items, distorting the results and reducing the accuracy and reliability of the assessment. Non-experts may also base assessments on subjective impressions of appearance or environmental conditions rather than on concrete background knowledge or data, which could lead to overly negative or positive evaluations (19, 23, 35). Disagreements among evaluators may arise during the conclusion process, making it difficult to reach a consensus (22, 24, 26). To mitigate these issues, it is essential to provide comprehensive training to tool users (16–18). This can be achieved through user manuals, workshops, and online training programs, which would help improve the accuracy and reliability of the assessments (4, 19, 26). Moreover, establishing remote support systems with experts and automated feedback mechanisms could help non-experts quickly resolve issues encountered during the evaluation process (21, 25, 26). By addressing these challenges, S-AWAG could be effectively utilized not only in small zoos but also in larger facilities, allowing a wider range of individuals to contribute to improving animal welfare.

### 4.5 Future potential and international application of S-AWAG

S-AWAG holds significant promise for standardizing animal welfare assessments in zoos. To fully realize this potential, it is crucial to consider how S-AWAG compares to international animal welfare standards and how it can be established and developed within South Korea. Additionally, for broader international application, it is important to explore how S-AWAG can evolve to align with the standards of different countries.

First, understanding both the strengths and challenges of S-AWAG is essential for its successful implementation at the national level. Designed to allow non-experts to easily participate in animal welfare assessments, S-AWAG complements traditional expert-driven methods with a more accessible approach. The inclusion of non-experts enhances the diversity and inclusivity of evaluations and ensures consistency, even in smaller zoos with limited resources (19, 24, 25). However, turning this potential into reality will require systematic educational programs, resource support, and policy adjustments at the governmental level. Legal support is another key factor for the successful adoption of S-AWAG. The Act on the Management of Zoos and Aquariums, enacted in 2016 (See footnote 8), legally permitted the participation of non-experts in animal welfare assessments. However, a 2022 amendment (See footnote 9) to this law strengthened the requirements for regular welfare assessments and oversight, with a particular focus on the involvement of professionals, particularly veterinarians. This legal shift suggests potential limitations to the participation of non-experts. Therefore, rather than solely focusing on expanding non-expert involvement, it may be more effective to position S-AWAG as a supplementary tool. By providing appropriate education and resources, S-AWAG can be used effectively alongside expert-driven assessments, aligning better with legal requirements and improving its overall functionality (21–24).

While expert-led evaluation systems remain crucial in animal welfare assessments, integrating non-expert participation as a complementary approach can provide several benefits (21, 22, 24). For instance, non-experts could utilize basic tools like S-AWAG during initial assessments or for routine monitoring of animal welfare conditions. When more complex welfare improvements or in-depth evaluations are needed, experts can intervene, creating a hybrid model that enhances both the reliability and effectiveness of assessments (24, 25, 28). Such a collaborative system could streamline large-scale assessments, enable quicker responses, and contribute to both the efficiency and accuracy of animal welfare assessments. Furthermore, involving the public can increase transparency in zoo operations and boost societal acceptance, ultimately fostering public trust in zoos and driving ongoing welfare improvements (23, 26, 29).

S-AWAG’s simplicity and flexibility also make it particularly useful for developing countries or smaller zoos with limited resources (15, 28, 31). These characteristics suggest that S-AWAG could be widely applied both internationally and within smaller zoos in South Korea. In resource-constrained settings, regular expert-led evaluations may be impractical (8, 28, 30). In such cases, S-AWAG, which can be easily utilized by non-experts, could enable routine and efficient welfare assessments, helping zoos maintain consistent standards of animal welfare in various environments (35, 47, 49). However, in larger zoos that follow stringent welfare standards established by international organizations like the AZA (See footnote 6) or EAZA (See footnote 7), S-AWAG may not serve as a complete replacement for existing specialized tools. These institutions often rely on highly advanced assessment methods (8, 14, 39). Nonetheless, S-AWAG could still play a valuable role as a supplementary tool in such environments. It could provide additional data to support advanced standards defined by experts while incorporating on-site observations and public input, resulting in a more comprehensive and multifaceted evaluation (21, 22, 24). In this way, S-AWAG could contribute to more robust welfare assessments, even in large zoos.

## 5 Conclusion

In conclusion, while S-AWAG is a valuable tool for animal welfare assessments, its limitations—including potential omissions of key welfare factors, issues with representativeness and reproducibility due to variations in observation conditions and assessor expertise, regional and species-specific constraints, and challenges when used by non-experts—should be addressed through continuous improvements. Enhancing the tool by incorporating qualitative measures, standardizing methodologies and equipment, and providing comprehensive training can ensure that S-AWAG remains effective and reliable across diverse settings. Future studies should aim to standardize observation conditions, thoroughly document methodological details, and expand the sample size and geographic scope to enhance the reliability and generalizability of welfare assessments using S-AWAG.

S-AWAG holds significant potential to become a valuable assessment tool across various settings, including developing countries, resource-limited zoos, and locations where expert availability is scarce. By enhancing its international recognition and applicability, S-AWAG could position South Korea as a leader in animal welfare practices. Ultimately, S-AWAG holds the potential to become a globally recognized tool for assessing and improving animal welfare.

## Data Availability

The raw data supporting the conclusions of this article will be made available by the authors, without undue reservation.

## Footnotes

^1^An Act to Authorize the Secretary of Agriculture to Regulate the Transportation, Sale, and Handling of Dogs, Cats, and Certain Other Animals Intended to Be Used for Purposes of Research or Experimentation, Pub. L. No. 89–544, 80 Stat. 350 (1966). Available at: https://www.govinfo.gov/app/details/STATUTE-80/STATUTE-80-Pg350-2 (Accessed 17 March 2024).

^2^République Française (n.d.). Available at: https://www.legifrance.gouv.fr (Accessed 17 March 2024).

^3^European Convention for the Protection of Animals Kept for Farming Purposes (1978). Available at: https://eur-lex.europa.eu/legal-content/EN/TXT/?uri=celex%3A21978A1117(01) (Accessed 17 March 2024).

^4^UK Government (1981). Zoo Licensing Act 1981. Available at: https://www.legislation.gov.uk/ukpga/1981/37 (Accessed 17 March 2024).

^5^Australian Government (n.d.). Department of Agriculture, Water and the Environment. Available at: https://www.awe.gov.au (Accessed 17 March 2024).

^6^Association of Zoos and Aquariums (n.d.). Available at: https://www.aza.org/ (Accessed 17 March 2024).

^7^European Association of Zoos and Aquaria (n.d.). Available at: https://www.eaza.net/ (Accessed 17 March 2024).

^8^Ministry of Environment (2016). Available at: https://www.law.go.kr/LSW/eng/engLsSc.do?y=0&x=0&menuId=1&query=animal#liBgcolor15 (Accessed 17 March 2024).

^9^Ministry of Environment (2022). Available at: https://www.law.go.kr/LSW/eng/engLsSc.do?y=0&x=0&menuId=1&query=animal#liBgcolor15 (Accessed 17 March 2024).

^10^Joongang Daily. (2022, October 18). Animal protection law gets long-awaited overhaul, but advocates worry about loopholes. Joongang Daily. Available at: https://koreajoongangdaily.joins.com/2022/05/29/culture/features/korea-animal-protection-act-animal-abuse/20220529151155380.html.

^11^Lee, J. (2023, June 14). Gimhae zoo under fire for animal cruelty. The Korea Herald. Available at: https://www.koreaherald.com/view.php?ud=20230614000672.

^12^Ministry of Environment (2020). The status of small-scale private zoos in Korea. [Unpublished Report].

^13^Maeil Newspaper (2021, February 8). “Fundamental measures needed to prevent animals from starving to death in zoos.” Available at: https://www.imaeil.com/page/view/2021020818001386358 (Accessed 17 March 2024).

^14^Joongang Daily. (2022, October 18). Animal protection law gets long-awaited overhaul, but advocates worry about loopholes. Joongang Daily. Available at: https://koreajoongangdaily.joins.com/2022/05/29/culture/features/korea-animal-protection-act-animal-abuse/20220529151155380.html.

^15^Giljam, P. (2018, August 13). Poor animal welfare in zoos & how Wild Welfare is supporting improvements. Zoospensefull. Available at: https://zoospensefull.com/2018/08/13/poor-animal-welfare-in-zoos-how-wild-welfare-is-supporting-improvements-by-wild-welfare/.

^16^Yeung, J., & Jo, Y. (2024, January 11). Raccoons, foxes, meerkats: South Korea clamps down on controversial animal cafes. CNN. Available at: https://edition.cnn.com/travel/south-korea-animal-cafes-intl-hnk-dst/index.html.

^17^World Animal Protection. (n.d.). About us. World Animal Protection. Available at: https://www.worldanimalprotection.org/about-us.

^18^Farm Animal Welfare Council. (1979). Five freedoms. Available at: https://web.archive.org/web/20210516042045/https://assets.publishing.service.gov.uk/government/uploads/system/uploads/attachment_data/file/725803/Farm_Animal_Welfare_Committee.pdf.

^19^Ministry of Environment (2022). Status of Zoo Registration. Available at: https://www.me.go.kr/home/web/policy_data/read.do?menuId=10259&seq=8085 (Accessed 17 March 2024).
