# Supplementary material for: Simplifying the Animal Welfare Assessment Grid for enhanced accessibility
Source: Front Vet Sci. 2024 Nov 19;11:1459560. doi: 10.3389/fvets.2024.1459560 (PMC11611830; doi:10.3389/fvets.2024.1459560)
Supplement: Supplementary file 1 [file Table_1.docx]

|  | General condition | Clinical assessment | Fecal | Activity | Food & water intake | Abnormal behaviours |
| --- | --- | --- | --- | --- | --- | --- |
| Score | Weight, overall appearance, fur/feather condition | Injury, hair loss, vomiting, other clinical symptoms | Fecal condition present in more than 50% of cases | Level of activity | Feeding, drinking, hunger, and thirst | Self-harm, vomiting, stereotyped behavior |
| 1 | Weight within normal range, good condition and fur/feather condition | No clinical symptoms | Shaped well | Normal | Normal | None |
| 3 | 10% or less over/under | Minor illnesses and injuries can be treated within a short period of time | Soft but shaped | Slight increase/decrease | Slightly reduced intake, hunger pangs for 2-3 days | Slight to medium frequency, short duration, no damage |
| 5 | 20% or less over/under | Moderate illness or injury, able to recover after long-term treatment | Loose | Significant increase/decrease, but recoverable (estrus, etc.) | Reduced intake for 2 days, reported hunger for 6-7 days | Moderate to high frequency, slightly persistent, no damage |
| 7 | 30% or less over/under | Moderate illness or injury, difficulty recovering | Diarrhea | Significant increase/decrease and persist for a long time | Reduced intake, reported hunger for more than 9 days | High frequency, quite a bit of time, damage noted |
| 9 | Over/under by 30% or more | Chronic/acute illness or serious injury, difficulty recovering | Watery diarrhea | Lethargy/hyperactivity | Thirst, dehydration, or loss of appetite for more than 1-2 days | High frequency, substantial time, wounds or extensive hair loss |
| 10 | Over/under by 30% or more, Longer than 21 days | Serious injury/illness or severe welfare problems, unrecoverable | Bloody or mucous | Complete lethargy (no movement, minimal movement) | Loss of appetite, dehydration for more than 3 days (1 day for birds) | High frequency, most of free time, severe damage (large cuts, missing toes) |

**Supplementary Table 1. Simplified Animal Welfare Assessment Grid: Welfare factors for Health Sector**

**Supplementary Table 2. Simplified Animal Welfare Assessment Grid: Welfare factors for Health Sector**

|  | Housing | Group size | Furniture & enclosure design | Accessibility | Surrounding situation | Visitors |
| --- | --- | --- | --- | --- | --- | --- |
| Score | enclosure size, temperature, humidity, lighting, ventilation, sufficient shelter & shade, environmental material & substrate (e.g., straw litter), drainage, UV, suitability of enclosures, noise & vibration, and hygiene. | Close to natural group size, appropriate size for enclosure, group structure is appropriate | Items such as branches, plants, hiding places, shelters, etc. are sufficient to represent the natural behavior of the animal. | Access to all of  enclosure | Animal translocation, zoo transitions, construction, and visitor disturbance | Visitor number, noise level, adverse interaction |
| 1 | 0-1 criteria scored 7 or more | suitable | Suitable | Freedom of movement | None | No visitors |
| 3 | 2-3 criteria scored 7 or more | slightly different group structure or slightly more or fewer animals | Natural behaviors can be expressed however the options provided for this are minimal | Shut in/out good/large  sized enclosure for one day | Minor disturbance from birds or construction | <50 visitors per hour, less noise |
| 5 | 4-6 criteria scored 7 or more | Group structures differ or group density is high | One type of natural  behavior is limited | Shut in/out  medium-sized  enclosure for part~1 day | Moderate disruption due to construction, etc. | 50–100 visitors per hour,  Increased noise or disruptive activity |
| 7 | 7-9 criteria scored 7 or more | Group structures differ significantly or group size is quite large or small | Numerous natural behaviors is limited | Shut in/out enclosure for two to seven days | Obvious disturbances such as construction, multiple birds, etc. | 50–100 visitors per hour, Loud noises or lots of distracting activity |
| 9 | 10 criteria scored 7 or more | Improper group structure and high density | Natural behaviors are very limited | Shut into small indoor/  outdoor enclosure for part~1 day | Disrupted by construction for more than 7 days, moved to a new enclosure | >100 visitors per hour, Moderate noise |
| 10 | 11-12 criteria scored 7 or more | No natural grouping at all (solitary housing of social animals, severe overcrowding) | The options are not  provided | Shut into small indoor/ outdoor enclosure for more than one day or medium/large enclosure for more than seven days | Moving to a new enclosure, constant external distractions | >100 visitors per hour, Loud noises or lots of distracting activity |

**Supplementary Table 3.** **Housing factor’s 12 criteria**

|  | Enclosure size | Temperature | Humidity | Lighting | Ventilation | Sufficient shelter & Shade |
| --- | --- | --- | --- | --- | --- | --- |
| Score | Enough space, at least a decent size (not restricted in movement), and social life (able to live individually without interfering with each other) | Proper temperature | Proper humidity | Proper and enough lighting | Air circulation | Providing shelter and shade where individual animals can rest appropriately |
| 1 | Behaviorally, socially, and individually free enough | Optimal temperature for animal health | Optimal humidity for animal health | Adequate and sufficient natural light close to the habitat (providing continuous open space) | Provide continuous open space with continuous ventilation, such as outdoor enclosures | There is enough comfortable shelter and shade for all animals to use whenever they need it, and there is a choice. |
| 3 | As large as possible for behavioral and social life | Animals can be kept healthy (proper temperatures as per husbandry manual) | Animals can be kept healthy (proper humidity as per husbandry manual) | Sufficient natural light (limited by obstructions from ceilings, etc., but sufficient natural light is available) | Ventilation is provided at the appropriate level as per the manual (no health problems for animals) | Provide comfortable shelter and shade for each animal to use when needed. |
| 5 | Only get the location of individuals behaviorally, socially, and individually | Possible to maintain the life of animals (minimum standards under animal laws, approximately 30% of the husbandry manual) | Possible to maintain the life of animals (minimum standards under animal laws, approximately 30% of the husbandry manual) | Provides the necessary natural or artificial light, no problems with breeding such as sunlight duration | Animals are only allowed to maintain their life (minimum standards under animal law, approximately 30% of the husbandry manual) | Provide minimal shade and shelter to avoid extreme heat and cold. |
| 7 | Behaviorally and socially viable, but lacking in individual location (1-2 individuals appear spatially anxious) | Temperature is not suitable for some time of the year (within 2 weeks), 1-2 animals have health or activity problems due to temperature issues | Humidity is not suitable for some time of the year (within 2 weeks), 1-2 animals have health or activity problems due to humidity issues | Provide minimal natural or artificial light, and problems with biological cycles such as reproduction or appetite | Poor ventilation for more than 6 hours/day | Shelter and shade are available, but 1-2 individuals are unable to use them. |
| 9 | Behaviorally able to live, but social herding and lack of individual space (frequent fighting, >50% animal anxiety) | Temperature is not suitable for some time of the year (2-4 weeks), many animals have health and activity problems due to temperature issues | Humidity is not suitable for some time of the year (2-4 weeks), many animals have health and activity problems due to humidity issues | Some natural light (normal glass that cannot pass through UV), provides normal light, but does not pose a major health risk to the animals | Poor ventilation most of the day (1-2 individuals showing health problems) | Shelter and shade are available, but only some individuals can use them. |
| 10 | Inability to live a behaviorally, socially, or personally appropriate life | Temperature is not right for more than a month out of the year. Some animals die or become ill due to temperature issues. | Humidity is not right for more than a month out of the year. Some animals die or become ill due to humidity issues. | Some natural light (normal glass that cannot pass through UV) provides general light that is not suitable for animals, causing health problems for animals. | Poor ventilation most of the day (causing illness or death) | No shelter or shade |

**Supplementary Table 3.** **Housing factor’s 12 criteria (2)**

|  | Environmental material & Substrate (e.g., straw litter) | Drainage | UV | Suitability of enclosures | Noise & Vibration | Hygiene |
| --- | --- | --- | --- | --- | --- | --- |
| Score | Providing appropriate environmental materials, flooring, etc. | Smooth drainage | Provide sufficient UV to the animals that need it | Expose location to visitors or other animals | Noise or vibration caused by equipment, construction, or visitors | Hygiene and maintenance conditions |
| 1 | The floor and other materials of the animal enclosure are appropriate and provide a sufficient variety of necessary substrates (such as bedding). | The floor can dry within an hour as it has sufficient drainage. | Provide sufficient natural lighting and UV light to all | There are multiple spaces where animals can stay comfortable without being exposed to other animals or visitors (optional) | No inappropriate noise at all | A pleasant environment through regular cleaning and maintenance (at least twice a day) |
| 3 | Provide adequate animal flooring and other building materials, but provide the minimum necessary substrate ( such as bedding). | Drainage is good, allowing the floor to dry within 3-4 hours (without animals present, using a fan). | Provide UV lights to all | There is a space where animals can be comfortable without being exposed to other animals or visitors. | Noise is present occasionally (within 2 hours), but animals adapt and do not become stressed. | Completely eliminate the cause of diseases such as bacteria through regular cleaning and management (at least once a day), remove feces within 1 hour. |
| 5 | Some of the flooring and other materials used in the animal enclosure are inadequate or do not provide the necessary substrate, but there is no significant health problem for the animals. | No clogged drain | Provide minimum UV light to animals in need | An environment where the animal is exposed to other animals or visitors, but can hide (no stress to the animal) | Noise exists for more than 8 hours/day. No signs of disease due to stress are observed. | Regular cleaning and maintenance, no risk factors in the animal house |
| 7 | The floor and other materials of the animal enclosure are inadequate or do not provide any substrate at all. (Health and activity problems in 1-2 animals) | Poor drainage, with standing water on less than 10% of the floor for more than 8 hours (without animals present) | Provides minimum UV lighting for animals in need, but provides it in some spaces, making it difficult for 1-2 individuals to use. | More than 180 degrees of exposure to other species of animals or visitors | Presence of noise that causes a stress response of more than 8 hours for more than 1 week | Cleaning and maintenance to prevent serious contamination 2-3 times a week (fecal contamination within 10% of the animal enclosure) |
| 9 | Inadequate animal floor and other construction materials. No substrate provided (more than 50% of animals have health or activity problems) | Poor drainage, with standing water on less than 20~30% of the floor for more than 8 hours (without animals present) | Insufficient UV is provided to animals in need or more than 50% of the animals cannot use it. | More than 270 degrees of exposure to threatening (predatory) animals or visitors, with some space to hide behind the back | Noise that causes a stress response of more than 8 hours within 1 month | Cleaning and maintenance once a week, less than 50% of the animal enclosure is contaminated with feces |
| 10 | The animal enclosure floor and other construction materials are all unsuitable, and there is no substrate provided. (Some animals have died or become ill due to this problem.) | Poor drainage, with standing water on less than 40~50% of the floor for more than 8 hours (without animals present) | Animals that do not receive the UV they need, or there are animals that develop related diseases such as rickets, osteoporosis, and loss of appetite, or animals that die | 360 degrees of exposure to threatening (predatory) animals or visitors | If noise exceeds 8 hours and continues for more than a month, the animal feels severe stress and shows stress-related diseases. | Cleaning management is not properly performed, such as once every 2-3 weeks or once a month. More than 50% of the animal enclosure is contaminated, and animals are contaminated with feces. |
